# Assessing Social Engagement in Heterogeneous Groups of Zebrafish: A New Paradigm for Autism-Like Behavioral Responses

**DOI:** 10.1371/journal.pone.0075955

**Published:** 2013-10-08

**Authors:** Hans Maaswinkel, Liqun Zhu, Wei Weng

**Affiliations:** Research and Development, xyZfish, Ronkonkoma, New York, United States of America; Tulane University Medical School, United States of America

## Abstract

Because of its highly developed social character, zebrafish is a promising model system for the study of the genetic and neurochemical basis of altered social engagement such as is common in autism and schizophrenia. The traditional shoaling paradigm investigates social cohesion in homogeneous groups of zebrafish. However, the social dynamics of mixed groups is gaining interest from a therapeutic point of view and thus warrants animal modeling. Furthermore, mutant zebrafish are not always available in large numbers. Therefore, we developed a new paradigm that allows exploring shoaling in heterogeneous groups. The effects of MK-801, a non-competitive antagonist of the glutamate N-methyl-D-aspartate (NMDA) receptor, on social cohesion were studied to evaluate the paradigm. The drug has previously been shown to mimic aspects of autism and schizophrenia. Our results show that a single MK-801-treated zebrafish reduced social cohesion of the entire shoal drastically. Preliminary observations suggest that the social dynamics of the shoal as a whole was altered.

## Introduction

Zebrafish is a highly social species [1] that plays an increasing role as a model organism for the study of neuropathological diseases that affect social functions, such as autism and schizophrenia [2], [3]. Zebrafish usually swim in shoals [4] and social cohesion (as determined by the distances between the zebrafish) and shoal preferences can be affected by a variety of environmental factors such as signals of danger [5] or food availability [6]. Using social network techniques, Vital and Martins [7] have shown that some ‘key’ individuals influence the behavior of the shoal as a whole more than do ‘non-key’ individuals. Interesting is also the occurrence of social learning and behavioral traditions [8]. Social interactions in zebrafish have been investigated in the context of antagonistic behaviors and social hierarchies [9], mating rituals [10] and social preferences [11]. The effects of drugs on social behaviors in zebrafish have been explored in several studies [12]–[15].

MK-801, a non-competitive antagonist of the glutamate NMDA-receptor, affects social and non-social behaviors in animal models and is applied to simulate aspects of autism and schizophrenia [16]. Indeed, dysregulation of the balance between excitatory glutamate and inhibitory γ-amino-butyric acid (GABA) neurotransmission has been suggested to be linked to ASD (autism spectrum disorder) [17]. Furthermore, it has been shown that glutamate/glutamine was reduced in the basal ganglia of individuals diagnosed with ASD and that this reduction was correlated to increased communication impairment [18]. One of the genes implicated in ASD is GRIN1 which encodes the NMDA-receptor subunit zeta-1 [19]. Interestingly, MK-801 attenuates social preferences in mice [20], reduces social investigative behaviors [21] and increases social withdrawal in rats [22]. It also causes hyperactivity in rats [21], circling behavior in mice [23], and reduction of prepulse inhibition in rats [24]. In mice, it creates auditory electrophysiology disruptions that are reminiscent of those found in autism [25]. Behavioral and pharmacological actions of NMDA seem to be conserved in zebrafish [26]. MK-801 reduces the preference of zebrafish for a group of stimulus zebrafish [14] and disrupts shoaling [27]. Furthermore, it impairs memory performance in the plus maze associative learning paradigm [28], the inhibitory avoidance task [14], [29], and the y-maze memory task [30]. It has a tendency to increase erratic movements [31], it increases circling behavior [32], time spent at the top of the tank [27], and locomotion [26].

In this study we introduce the heterogeneous shoaling paradigm in which only one of the members of the shoal is characterized by a social engagement deficit (which could be induced by a drug, be the result of genetic factors or of experimental manipulations). Here we use MK-801 to evaluate the paradigm. The rationale for using MK-801 instead of mutant zebrafish is that the drug simulates autism-like symptoms well (see above), whereas most zebrafish autistic-gene mutations are not yet catalogued well enough to determine where on the spectrum of ASD they are located and thus how well they are suited for the current purpose.

In the first experiment homogenous shoals of zebrafish were tested, i.e. shoals in which all members were pre-exposed to MK-801. The main purpose of this experiment was to determine the effective dose to significantly alter both social and non-social behaviors. The second experiment explored the effects of MK-801 on heterogeneous shoals, i.e. shoals in which only one of its members was pre-exposed to the drug. The rationale for this new paradigm was twofold. First, when investigating altered social engagement, an interesting question is how certain individuals affect the dynamics of the group as a whole [33]. This social network approach is slowly gaining attention in zebrafish research [7]. In children diagnosed with ASD, interactional interventions involving parents, teachers, peers [34], or pets [35] seem to be promising. Therefore, the mixed-group approach in animal models of autism is of great interest. Second, it can be difficult to obtain a large enough number of mutants for the traditional homogeneous shoaling paradigm. Typically 80–100 individuals are needed for every experimental condition when studying shoals consisting of four zebrafish [15]. If the mutants have reduced viability or the goal is to screen large numbers of candidates (when applying forward genetics) it is often difficult or unpractical to obtain enough zebrafish. Thus it is crucial to know whether the heterogeneous shoaling paradigm can be applied as an alternative.

## Materials and Methods

### Subjects

Six-month old female zebrafish (*Danio rerio*) of an unspecified (‘short-fin’) wild-type strain were purchased from Aquatica Tropicals, Inc (Plant City, Fl, US). In total 460 zebrafish were used. They were acclimated to the laboratory conditions for at least 4 weeks in 38-liter aquariums. Water temperature was equal to room temperature (approx. 23°C). Light regimen: 14 hrs lights on (6 am–8 pm), 10 hrs lights off. Zebrafish were fed three times a day: at 8 am Tetra tropical flakes; at 12 pm live brine shrimp larvae; at 3 pm Tetra tropical flakes. On the days of the experiments, the zebrafish were fed flakes only at 8 am. Immediately after the recordings, the zebrafish were euthanized with 300 mg/L tricaine methanesulfate (MS-222). All experiments were conducted in accordance with IACUC guidelines and this study was approved by xyZfish IACUC.

Although in the wild, zebrafish shoals can be of any sex composition [1], we decided not to mix sexes (similar as in [15], [36]). Recent studies [37], [38] confirm our view that male and female zebrafish show clearly distinct behavioral patterns. We used only females as to avoid complicating social cohesion by occurrences of antagonistic behaviors. Whereas in males aggression often emerges very quickly, in females it is delayed for up to 24 hrs [9]. Before using the new paradigm with male zebrafish or mixed groups, it would be advisable to assess the effects of MK-801 on aggression and mating. Finally, no pretest was performed to separate key from non-key individuals [7]. Since the fish were selected at random from their home tanks and the groups were relatively large (18–20 quadruplets per experimental condition), we assume that the results were not biased by social ranks.

### Drugs

(+)-MK-801 hydrogen maleate (M107) and MS-222 (A5040) were purchased from Sigma. A stock solution (about 0.6 mM) of MK-801 (using water as vehicle) was made once per week and kept in the refrigerator at about 4°C.

### Apparatus

**Figure 1:**
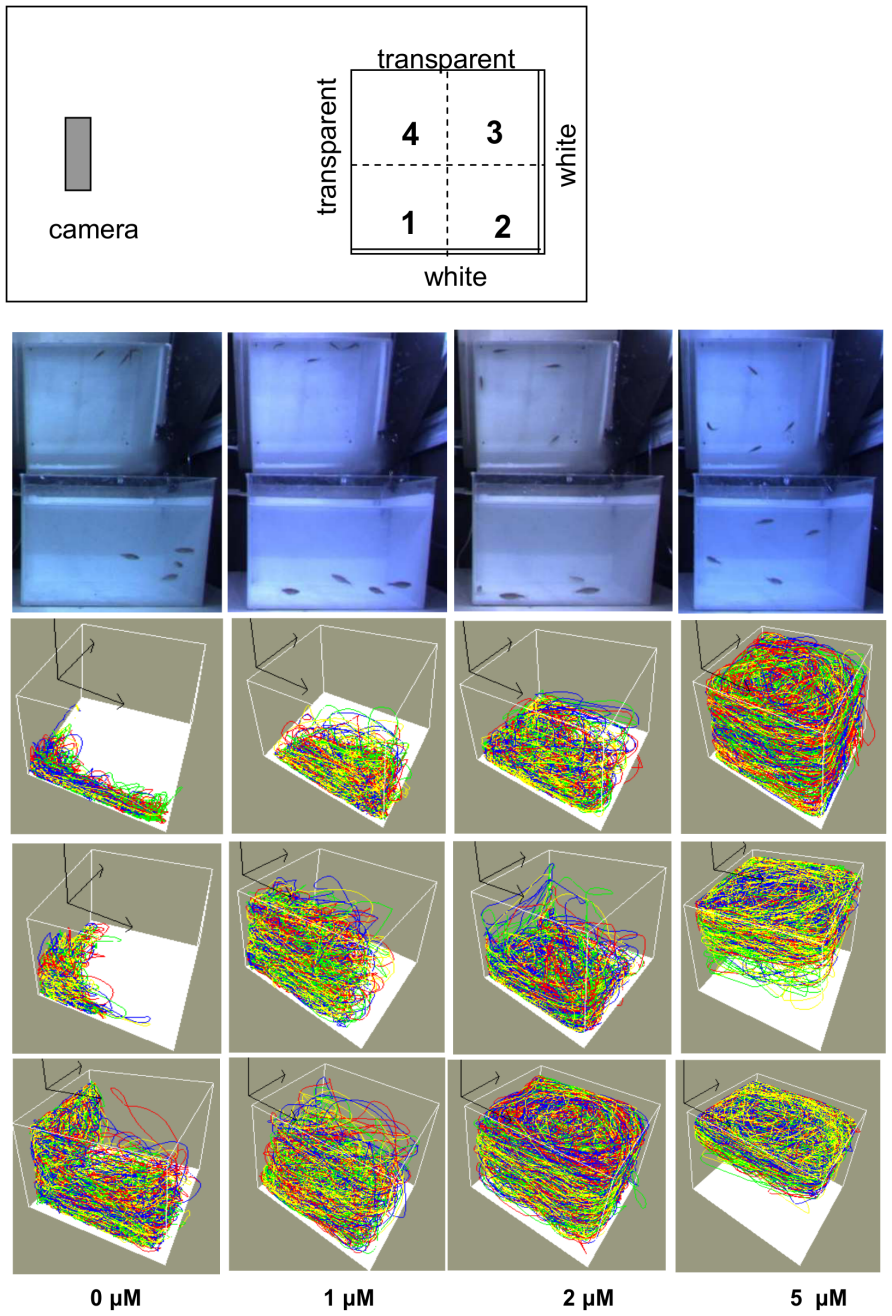
Layout of apparatus. Sample frames and trajectories for the homogeneous shoals in experiment 1. The top panel represents the layout of the observation chamber and the positioning of the observation container. The locations of the quadrants, the transparent and white walls are also indicated. One typical sample frame and three representative trajectories are shown for every concentration of MK-801 for the homogeneous shoals. Column 1∶0 µM, column 2∶1 µM, column 3∶2 µM, column 4∶5 µM. Note that the sample frames show the front and (on the mirror) the top view. The different colors represent different zebrafish. Note however, that tag-swapping between zebrafish occurs. The origin of the three arrows is located to the left of the camera (for orientation). Note that with increasing drug concentration, the distance between the fish increases and the horizontal distribution becomes more homogeneous. At the highest concentration (5 µM) the fish swim on average closer to the water surface.

The apparatus [39]–[41] consisted of stackable observation compartments (length, 91 cm; width, 46 cm; height, 56 cm) closed by curtains. The transparent observation container (length, 25 cm; width, 25 cm; height, 18 cm; water level: 13.5 cm) was placed close to one side on the long axis with the camera (Bumblebee 2; Point Grey research Inc, Vancouver, Canada) on the other side (see Fig. 1 for layout). Above the container a mirror was suspended at an angle such that the top and front views could be recorded simultaneously. LED bars were suspended above water level in such a way that the illumination was more or less homogenous at water level (approximately 800 lux). The curtains were dark green. The bottom, the far and right walls (relative to the camera) of the containers were painted white to increase contrast. The frame rate for recording was approximately 40 frames per second (fps). The 3D-coordinates were extracted to calculate the trajectories and spatial allocations of the zebrafish. The position of every zebrafish was determined by its centroid (i.e. geometric center). The software corrected for light refraction.

### Procedure of Experiment 1: Homogeneous Shoals

At 9∶30 am, quadruplets of zebrafish were placed into 1.7-L containers holding 1 L conditioned water with either of the following MK-801 concentrations: 0, 1, 2 or 5 µM. The duration of exposure to water or MK-801 was 60 min (similar to [27]). At 10∶30 am, the quadruplets were transferred to the observation containers and the recording was started within 1–2 min (to let the water movements dissipate) and lasted 20 min. The assignment of the four experimental groups to the eight chambers used in this experiment was balanced across the ten experimental days to avoid biasing by potential (e.g. visual, magnetic, auditory) variations in the environment. Per group 20 quadruplets were tested. However, in the 5 µM-group, one fish jumped out of the container. Therefore, this quadruplet could not be analyzed. Thus, for 0 µM, n = 20; for 1 µM, n = 20; for 2 µM, n = 20; for 5 µM, n = 19.

### Procedure of Experiment 2: Heterogeneous Shoals

For this experiment, nine observation chambers were used. At 9∶30 am, twenty-seven zebrafish were placed in an 8-L container holding 6 L water, four or five (alternating per day) zebrafish were placed in 1.7-L containers containing water, and five or four (alternating per day) zebrafish were placed in 1.7-L containers containing 5 µM MK-801. At 10∶30 am, groups of three zebrafish from the 8-L container were transferred to each of the nine observation containers. Subsequently, one fish from one of the 1.7-L containers (either containing water or 5 µM MK-801) was added to every observation container (completing the quadruplets). The assignment of the latter two groups to the observation chambers was balanced over the four experimental days as to avoid biasing the experiment. Per condition (control: three-plus-one control zebrafish; MK-801: three-control/one-MK-801 zebrafish), 18 quadruplets were tested (for control group, n = 18; for MK-801 group, n = 18). The recording was started within 1–2 minutes after placing the fish into the apparatus (to let the water settle) and lasted 20 min.

### Data Analysis

Since the effect of MK-801 on social cohesion was the primary focus of this study, we calculated four different commonly-used parameters of social cohesion: inter-individual distance (IID), nearest neighbor distance (NND), farthest neighbor distance (FND) and shoaling index (SI). IID is the average of all the (six) distances between the four zebrafish. NND is the distance between any fish and its closest neighbor. FND is the distance between any fish and its farthest neighbor. The definition of SI is modified from Chivers et al. [42] and is assigned as follows: 1, when no fish is within one body length (we took an average body length of 32 mm) from any other fish; 2, when only two fish are within one body length from another fish; 3, when three fish are in a group which are chained by distances of less than one body length or when there are two groups of two fish that are within one body length from each other; 4, when all four fish are chained by distances of less than one body length. Note that although SI is a discrete variable, its value per quadruplet is averaged over all (about 48,000) frames per recording. Therefore it is statistically treated as a continuous variable. We used four measures of social cohesion, because we hypothesized that they might be differently affected by the presence of only one zebrafish treated with MK-801 (experiment 2) provided the behaviors of the other three zebrafish remain largely unchanged. For instance, IID is 50% determined by a zebrafish with aberrant behavior (it determines three of the total six inter-individual distances), but NND is only 25% determined by it.

To determine whether the distances between the fish were differentially affected by the heterogeneous composition of the shoal (experiment 2), the four components of NND were calculated per recorded frame and arranged from smallest to largest (NND_1,_ NND_2_, NND_3_ and NND_4_; note that by definition NND_1_ = NND_2_). If the zebrafish treated with MK-801 swims on average at a greater distance from the three control zebrafish which stay closer together (as the results from experiment 1 would suggest), then in most frames (of the about 48,000 frames per recording) NND_4_ is determined by the MK-801 zebrafish and NND_1–3_ by the control zebrafish. Thus, the four NND-components should then be differentially affected by the drug. On the other hand, if the control zebrafish adjust their inter-individual distances to the presence of the MK-801 zebrafish, then a direct assignment of NND_4_ to this latter zebrafish is not possible. This indirect method is applied, because the recording system does not consistently identify single zebrafish. Tag-swapping occurs relatively frequently, especially when four or more fish are recorded simultaneously. The analysis of the NND-components is limited in value and has no direct implication for the evaluation of the heterogeneous-shoaling paradigm per se (which is simply done by using any of the four main measures of social cohesion: IID, NND, FND, SI). However, it might provide an indication why the paradigm works (if it works). To further explore the possible effect of the MK-801 zebrafish on the social cohesion between the three control zebrafish, some visual observations were also reported.

Average distance from bottom was calculated as measure of vertical distribution. Distance from center and proportion of time spent in the quadrants were taken as measures of horizontal distribution. Fig. 1 shows the location of the quadrants. Note that the quadrants are qualitatively different in regard to the visual surroundings. Quadrants 1 and 3 are each lined by one transparent and one white wall. However, the transparent wall limiting quadrant 1 provides a view of a larger open space than does the one limiting quadrant 3. Quadrant 2 is limited by two white walls and quadrant 4 is limited by two transparent walls. Finally, total travel distance was determined.

For the multi-dose experiment 1, we performed Shapiro-Wilk tests for normality and Levene’s tests for equality of variances. If the distribution was normal and the variances were equal, we applied an ANOVA to test for overall drug effects followed by post-hoc Bonferroni tests. If those conditions were not met, we used a Kruskal-Wallis test for overall drug effects followed by post-hoc Dwass-Steel-Critchlow-Fligner tests. The posthoc tests were only applied to test the effects of the different concentrations of MK-801 relative to the control group. Since we tested four (groups of) independent variables (social distance in four variations, distance from bottom, horizontal distribution in two variations, and locomotion) we applied α = 0.05/4 = 0.0125. For the distribution over the four quadrants, we also performed chi-square tests (with α = 0.05) to assess whether in any of the conditions it was homogeneous. For experiment 2, the same statistical tests were performed with the appropriate adaptations: Mann-Whitney U-tests were used as non-parametric tests and post-hoc tests were obsolete.

## Results

Two experiments were performed. In the first experiment, exploring the homogeneous-shoal condition, every quadruplet (i.e. shoals of four individuals) was pre-exposed to 0, 1, 2 or 5 µM MK-801 for 60 min before recording its behaviors for 20 min. The goal was to find a dose for which both social behaviors (as measured by four distinct parameters of social cohesion) and non-social behaviors (in this case vertical and horizontal distribution and locomotion) were affected by the drug. In the second experiment, heterogeneous quadruplets were tested. In this case, only one of the four zebrafish in every quadruplet was pre-exposed to either 0 or 5 µM MK-801. The first question was whether changes in social cohesion were preserved under this condition and, if so, whether any of the four parameters for social cohesion was better suited for this purpose than others. The second question was whether the new paradigm allows isolating the social effects from the non-social effects of the treatment.

### Experiment 1: the Effects of MK-801 on Homogeneous Shoals

Fig. 1, second row, shows representative frames for every drug concentration. The social clustering of the zebrafish close to the front wall for the control group and the horizontal spreading-out, vertical high-level swimming combined with greater inter-individual distances for the 5-µM group were very characteristic. The configuration of the 1-µM group closely resembled that of the control group, albeit greater inter-individual distances were often seen. Frames of the 2-µM group resemble those of the 5-µM group. However, bottom-hugging as occurred in some of the quadruplets treated with 2-µM was very uncharacteristic for the 5-µM group, where top-swimming was more prevalent.

The trajectories provide more information (see Fig. 1, third to fifth rows). Characteristic for the 0-µM group is that the zebrafish spent most of their time in quadrants 4 and 1 (for location of the quadrants, see Fig. 1, top), with a preference for the former quadrant (which is lined by two transparent walls). Often, they stayed close to the bottom. In the 1-µM group, the zebrafish still showed a preference for the front wall, but were less skewed to quadrant 4. When increasing the concentration of MK-801 to 2 µM, the zebrafish spread more out over the horizontal plane, i.e. their preference for the front wall decreased. Nevertheless, they often stayed close to the bottom. However, some quadruplets seemed to have lost the preference for any horizontal or vertical location. This was also often observed in the 5-µM group. However, many of the quadruplets of this latter group showed a clear preference for the upper depth levels (close to the water surface).

**Figure 2:**
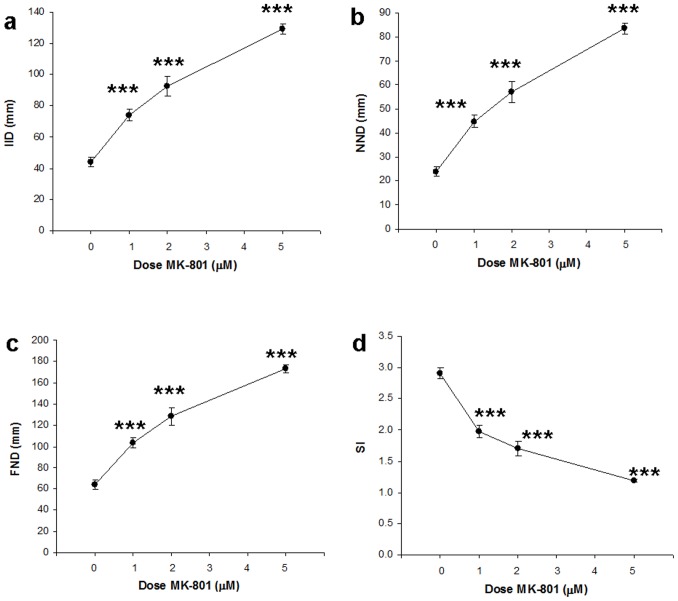
Social cohesion of the homogeneous shoals in experiment 1. All four measures of social cohesion show that with increasing drug concentration cohesion decreases. (**a**) IID, inter-individual distance, (**b**) NND, nearest neighbor distance, (**c**) FND, farthest neighbor distance, and (**d**) SI, shoaling index, are presented. Means ± SEMs are shown. Significant differences between MK-801 groups and controls: ***p<0.001. For 0, 1 and 2 µM MK-801, n = 20. For 5 µM, n = 19.

For social cohesion (Fig. 2a–d), we calculated IID, NND (nearest neighbor distance), FND (farthest neighbor distance) and SI (shoaling index). For IID, Kruskall-Wallis test revealed an overall drug effect (H = 58.2, p<0.00001). Post-hoc Dwass-Steel-Critchlow-Flinger tests showed that all three concentrations of MK-801 resulted in significant increases of IID (p<0.00005). For NND, Kruskall-Wallis showed that the overall drug effect was significant (H = 58.3, p<0.00001) and posthoc tests revealed that all three MK-801 concentrations increased NND in comparison to controls (p<0.00005). For FND the overall drug effect was significant (H = 57.2, p<0.00001). Post-hoc tests demonstrated that all three concentrations increased NND significantly (p<0.00005). For SI, Kruskal-Wallis test elicited an overall significant drug effect (H = 57.8, p<0.00001) and post-hoc Dwass-Steel-Critchlow-Fligner tests demonstrated that all groups significantly decreased relative to the control group (for 1 µM, p<0.00005; for 2 and 5 µM, p<0.00001). Thus according to all four parameters, social cohesion was decreased for all three concentrations of MK-801.

**Figure 3:**
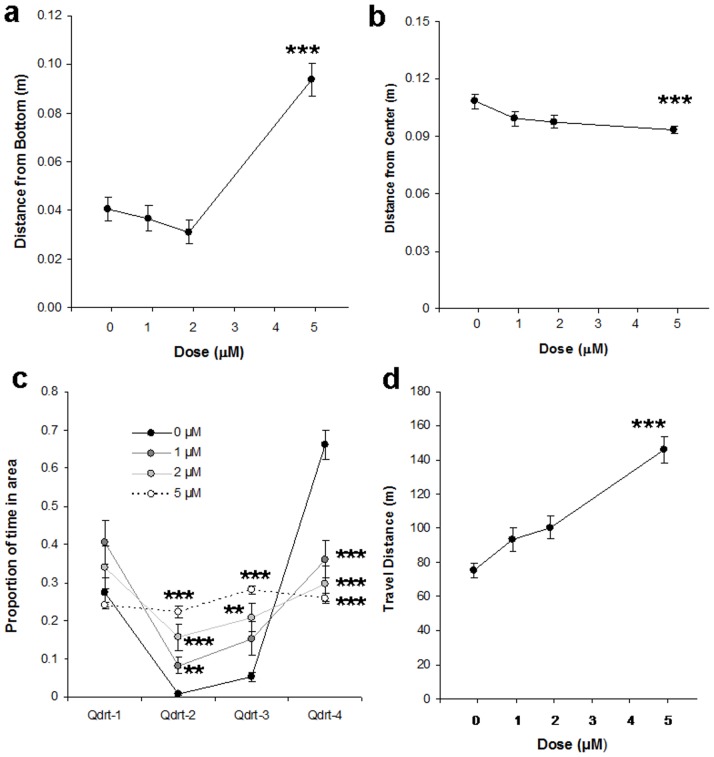
Non-social behaviors of the homogeneous shoals in experiment 1. (**a**) Distance from bottom increased significantly for 5 µM MK-801. (**b**) Distance from center decreased significantly for 5 µM MK-801. (**c**) The temporal distribution over the four quadrants became more homogeneous with higher concentrations of MK-801. (**d**) Travel distance was increased for 5 µM MK-801. Means ± SEMs are shown. Significant differences between MK-801 groups and controls: **p<0.005; ***p<0.001. For 0, 1 and 2 µM MK-801, n = 20. For 5 µM, n = 19.

For distance from bottom (Fig. 3a), Kruskal-Wallis test demonstrated that there was an overall drug effect (H = 33.4, p<0.00001) and post-hoc Dwass-Steel-Critchlow-Fligner tests revealed that only 5 µM MK-801 increased it significantly (p<0.001).

For distance from center (Fig. 3b), Kruskal-Wallis revealed an overall significant drug effect (H = 11.3, p<0.0125). Post-hoc tests showed that only 5 µM decreased it significantly (p<0.001).

For distribution of the zebrafish over the quadrants (Fig. 3c), no drug-effect was found for quadrant 1. For quadrant 2, there was an overall drug effect (H = 42.4, p<0.00001). The post-hoc tests showed that the time in quadrant 2 increased for 1 µM MK-801 (p<0.005), 2 µM (p<0.00005) and 5 µM (p<0.00005). Similarly, there was an overall drug effect for the time spent in quadrant 3 (H = 34.6, p<0.00001). Whereas 1 µM MK-801 did not significantly affect the time spent in quadrant 3, both 2 µM (p<0.005) and 5 µM (p<0.00005) increased it. Finally, there was an overall drug effect for the time spent in quadrant 4 (H = 33.3, p<0.00001) and all three doses of MK-801 decreased that time significantly (for 1 µM, p<0.001; for 2 µM, p<0.0001; for 5 µM, p<0.00001). Subsequent chi-square tests showed that the distributions over the four quadrants for the control and the 1-µM groups were not homogeneous. The 2-µM group was close to homogeneously distributed (p = 0.041), whereas the distribution of the 5-µM group was not distinguishable from a homogeneous distribution, i.e. no preference for any of the quadrants could be detected.

For travel distance (Fig. 3d), there was an overall drug effect (F [75, 3] = 22.5, p<0.00001). The post-hoc Bonferroni tests showed that 1 µM MK-801 did not significantly affect travel distance, 2 µM MK-801 had only a slight tendency to increase it (p = 0.031) and 5 µM MK-801 increased it significantly (p<0.00001).

### Experiment 2: the Effects of MK-801 on Heterogeneous Shoals

**Figure 4:**
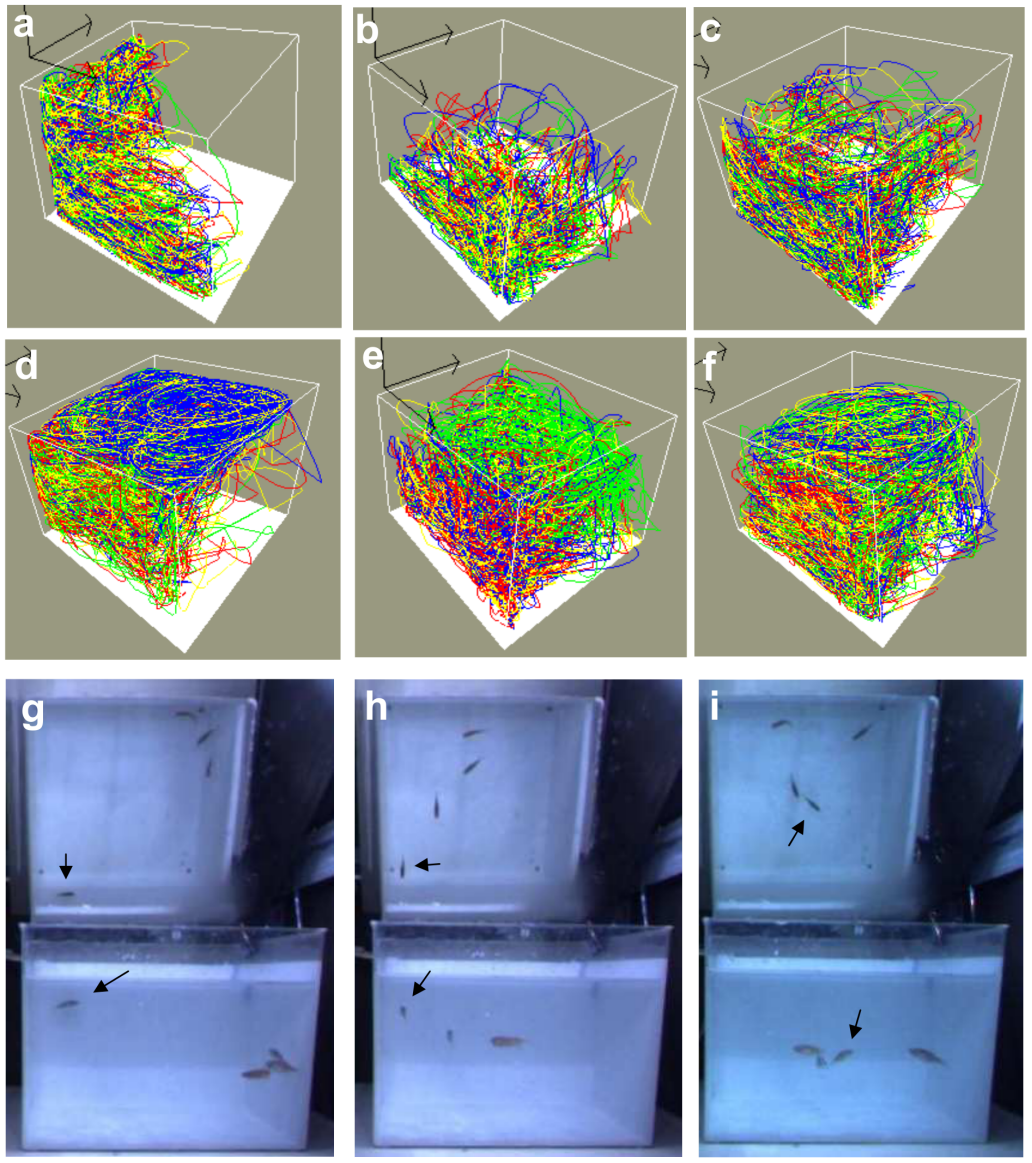
Example trajectories and frames for the heterogeneous shoals in experiment 2. The top row (**a–c**) shows trajectories of three control quadruplets and the second row (**d–f**) shows trajectories of three quadruplets consisting of three control zebrafish and one zebrafish treated with 5 µM MK-801. In panel (**d**) the trajectory of the zebrafish treated with MK-801 is presented in blue, in panels (**e**) and (**f**) they are presented in green. (**g**) The MK-801 zebrafish often swims far apart from the three control zebrafish. (**h**) The control zebrafish are frequently seen to follow the MK-801 zebrafish, (**i**) which often leads to increased distances between the three control zebrafish. The black arrows points to the MK-801 zebrafish.

The trajectories for the control group (Fig. 4a–c) were similar to those in experiment 1, whereas the trajectories for the experimental group (Fig. 4d–f), containing one zebrafish treated with 5-µM MK-801, were different from those for the 5-µM group in experiment 1. Whereas consistent swimming close to the top was often seen in the latter group (see examples in Fig. 1), it never occurred in the heterogeneous shoals for the trajectories of all four members of a shoal. However, in the cases that were manually corrected for tag swapping (i.e. those presented in Fig. 4d–f), it turned out that top-swimming was indeed performed by the 5-µM zebrafish.

Observations revealed that the three control zebrafish had a tendency to swim closely together whereas the zebrafish treated with 5 µM MK-801 often swam alone (Fig. 4g). Interestingly, however, the control zebrafish often follow the drug-treated zebrafish (Fig. 4 h) for short distances. This seemed to result in increasing the distances between the control zebrafish trios (Fig. 4i). No systematic scoring of this behavior was performed.

**Figure 5:**
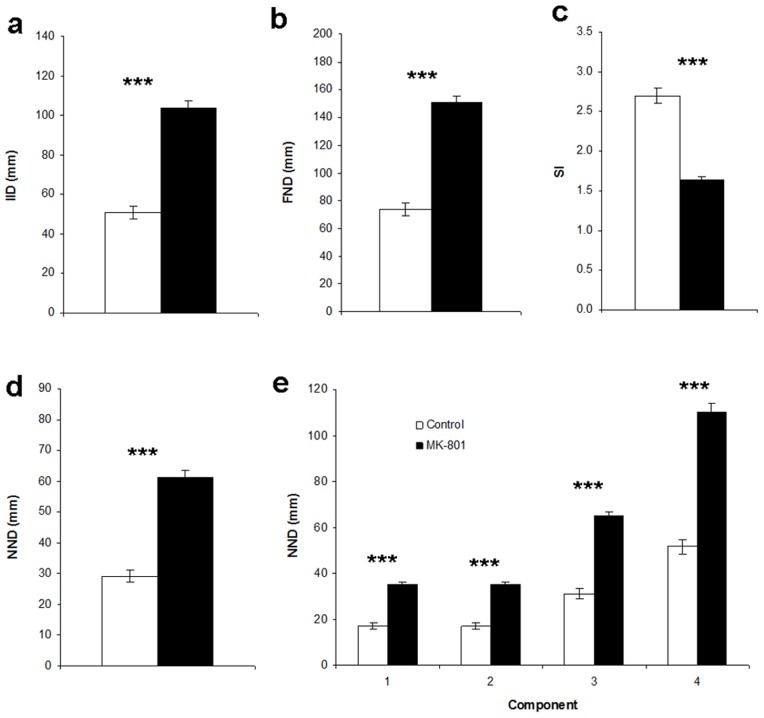
Social cohesion for the heterogeneous shoals in experiment 2. All four measures of social cohesion show that in the heterogeneous MK-801 group cohesion was decreased. (**a**) IID, inter-individual distance, (**b**) FND, farthest neighbor distance, (**c**) SI, shoaling index, are presented, (**d**) NND, nearest neighbor distance, (**e**) the four components of NND. White bars represent the control group (i.e. quadruplets consisting of four zebrafish pre-exposed to water), black bars represents the heterogeneous MK-801 group (i.e. quadruplets consisting of three zebrafish pre-exposed to water and one zebrafish pre-exposed to 5 µM MK-801). Means ± SEMs are shown. Significant differences between heterogeneous MK-801 and control groups: ***p<0.001. For control group, n = 18; for heterogeneous MK-801 group, n = 18.

According to all parameters, social cohesion was decreased by the presence of one zebrafish treated with MK-801 (Fig. 5a–e): IID (U = 25.6, p<0.00001), NND (U = 25.3, p<0.00001), its components NND_1,2_ (U = 23.7, p<0.00001), NND_3_ (U = 24.7, p<0.00001), NND_4_ (U = 26.3, p<0.00001) and FND (U = 26.0, p<0.00001) were significantly increased and SI (U [36] = 24.1, p<0.00001) was significantly decreased.

**Figure 6:**
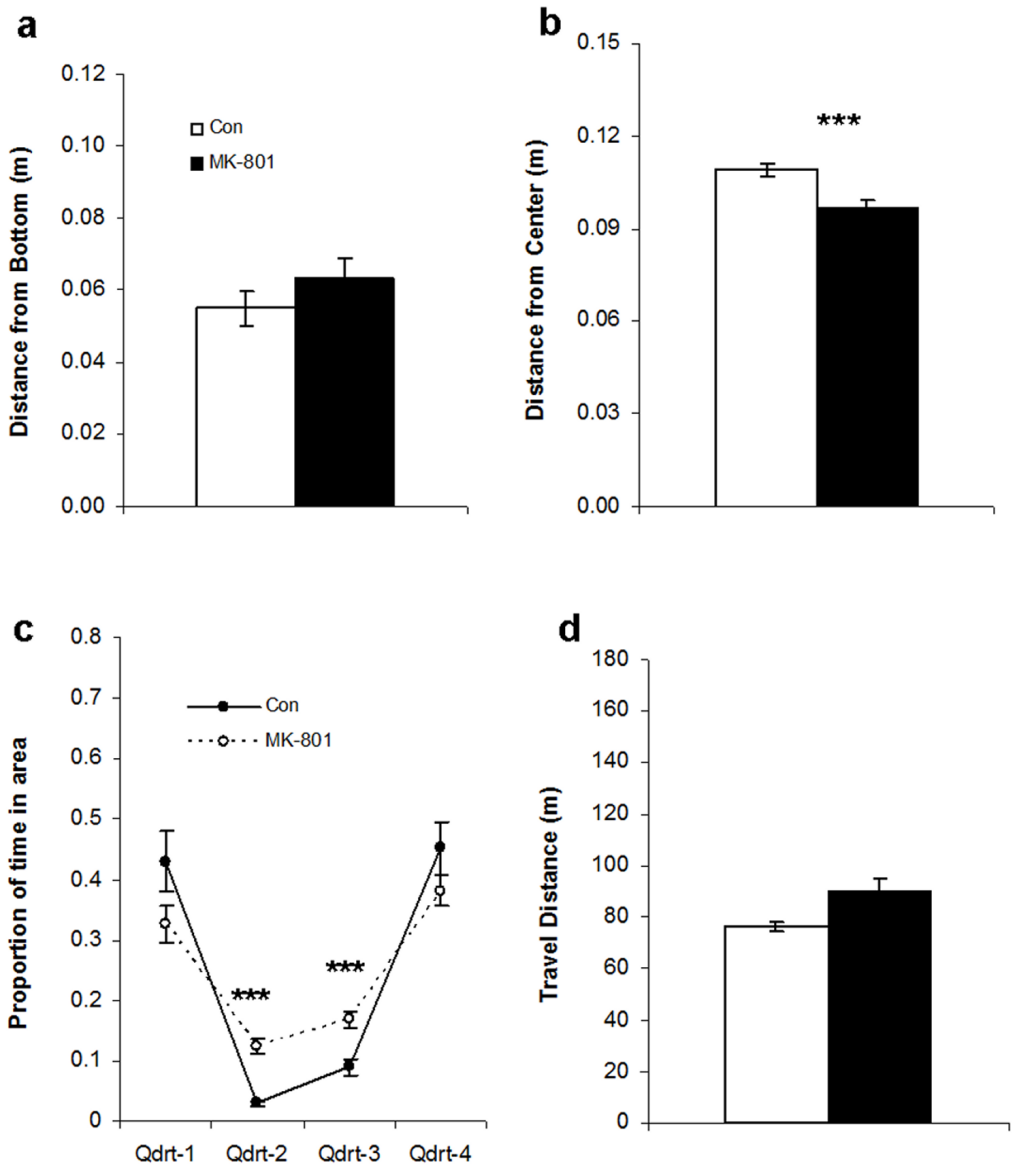
Non-social behaviors for the heterogeneous shoals in experiment 2. (**a**) Distance from bottom was not significantly different between groups. (**b**) Distance from center was significantly lower for the heterogeneous MK-801 group. (**c**) The heterogeneous MK-801 group spent more time in quadrants 2 and 3 than the control group. (**d**) Travel distance was not significantly increased for the heterogeneous MK-801 group. White bars represent the control group (i.e. quadruplets consisting of four zebrafish treated pre-exposed to water), black bars represents the heterogeneous MK-801 group (i.e. quadruplets consisting of three zebrafish pre-exposed to water and one zebrafish pre-exposed to 5 µM MK-801). Means ± SEMs are shown. Significant differences between heterogeneous MK-801 and control groups: ***p<0.001. For control group, n = 18; for heterogeneous MK-801 group, n = 18.

The average distance from bottom (Fig. 6a) for the shoal was not significantly affected by the presence of the MK-801-treated zebrafish, whereas distance from center (Fig. 6b) decreased (F [34], [1] = 14.2, p<0.001). The times spent in quadrants 1 and 4 (Fig. 6c) were not significantly affected by the presence of the MK-801 zebrafish. The experimental group spent more time in quadrant 2 (U = 15.0, p<0.00001) and in quadrant 3 (U = 51.0, p<0.0005) than the control group. Chi-square tests demonstrated that for both groups the distributions over the four quadrants were not homogeneous under either condition. Travel distance (Fig. 6d) did not significantly increase in the presence of the MK-801 zebrafish.

## Discussion

In experiment 1 (homogeneous groups), we found that MK-801 decreased social cohesion in a dose-dependent manner. This was observed for all measures: IID, NND, FND and SI. Non-social behaviors were also affected but mainly for the highest dose. Distance from bottom and travel distance were increased and distance from center was decreased for 5 µM MK-801. An interesting finding was that the preference for quadrants 4 and 1 in control zebrafish declined with increasing dose of MK-801 until it disappeared for 5 µM. In experiment 2 (heterogeneous groups), where only one of the four zebrafish was treated with 5 µM MK-801, the results were partially comparable to the homogeneous group treated with the same dose. Social cohesion decreased according to all four parameters. Distance from center was also decreased. However, distance from bottom was not affected at all. There was only a moderate shift to quadrants 2 and 3, which did not result in a homogeneous distribution as was seen for the same dose in the homogeneous-group experiment. Travel distance had only a tendency to be increased. Thus, the heterogeneous-group experiment replicated the social effects of MK-801 found in the homogeneous-group experiment better than the effects on spatial distribution and locomotion.

The interesting finding is that social cohesion was very significantly decreased when only one of the four zebrafish was treated with 5 µM MK-801. Our original purpose for using four measures of social cohesion was to determine which measure was best suited for this new paradigm. If we assume that only the single zebrafish treated with the drug showed decreased social affiliation, whereas the other three zebrafish did not change their behaviors amongst each other, then we would expect the parameters to be differently affected as follows: FND>IID>NND>SI. Basically, FND would depend mainly on the MK-801 zebrafish (i.e. the zebrafish that most likely swam at the greatest distance from the other three zebrafish), IID would depend for about 50% on it (because one zebrafish determines three out of the six components of IID), NND and SI would depend for about 25% on it. However, we found that none of the four parameters was better suited than any other for detecting decreased social cohesion in the heterogeneous groups. Thus, we have to assume that all four zebrafish changed their social behavior. Indeed, this is consistent with the fact that all four NND-components were (nearly equally, namely approximately 100%) increased. This was further supported by informal observations of the recorded movies: the control zebrafish often followed the MK-801 zebrafish (e.g. Fig. 4 h). We never saw that the drug-treated zebrafish reciprocated this behavior. Thus following bouts did not result in all four zebrafish clustering together as is common for control zebrafish. At the same time, following often resulted in the three control zebrafish spreading out more (e.g. Fig. 4i) which might explain the increase of the three first NND-components. Interestingly, when a control zebrafish came very close, the MK-801 zebrafish sometimes responded with small escape bursts. This was the only type of social ‘acknowledgements’ we could detect. Our impression was that the MK-801 zebrafish never approached another zebrafish or the general area (e.g. quadrant or depth level) occupied by another zebrafish except ‘coincidentally’. These observations are consistent with decreased social affiliation. Moreover, the only signs of social avoidance were the above mentioned small escape bursts. Social preference/avoidance tasks (using a mirror or a social preference test) should be performed to further explore the nature of social attraction and/or avoidance. We are aware that our observations are very preliminary and that future studies have to address these issues in more detail applying sound ethological methods.

With regard to the effects of MK-801 on social engagement, we can only compare the results of our first experiment (homogeneous shoals) with findings of other studies. Echevarria et al. [27] reported that after 60-min pre-exposure to 20 µM MK-801 social cohesion decreased. Lower doses were not tested. Since that study used an indirect method to determine social cohesion, it is impossible to quantitatively compare their data with our findings. Seibt et al. [14] found that 15-min pre-exposure to 5 µM MK-801 (other doses were not tested) decreased the preference of groups of five zebrafish to the side of the container that was adjacent to a tank containing 15 stimuli zebrafish. Sison and Gerlai [31] found that the preference of single experimental zebrafish for an adjacent tank containing five stimulus fish decreased in the presence of 100 µM MK-801. However, lower doses were ineffective. Furthermore, 30-min pre-exposure to the drug at any dose was ineffective. Finally, in another study [41] we pre-exposed shoals of four female zebrafish to 10 µM MK-801. The behavioral changes were similar to those described in the current study for 5 µM in experiment 1. Overall the reduction of social cohesion in the present study was predictable. However, because of the inconsistent dose-response data and the variations in experimental procedures across studies, the optimal drug concentration for our setup needed to be established (in experiment 1) before we could explore the new heterogeneous-shoaling paradigm (experiment 2).

The changes in spatial and kinematic parameters seen in experiment 1 could be explained in several ways. For instance, the 5-µM group swam at a greater distance from the bottom and closer to the center of the tank. Both observations are consistent with an anxiolytic hypothesis [43], [44]. This interpretation could diminish the value of MK-801 pre-exposure as a model for ASD, at least in respect to non-social behaviors, because heightened (social and non-social) anxiety is often reported in children and adolescents diagnosed with ASD [45], [46]. However, whereas both increased wall-hugging and increased bottom-dwell time during the first few minutes in an unfamiliar environment are well-described neophobic responses (which habituate within about 5 min and are susceptible to anxiolytic drugs) in zebrafish, those behaviors are not necessarily unequivocal signs of anxiety, especially not in a 20-min experiment (for further discussion see [15], [36]). Whether MK-801 increases or decreases anxiety is not solved (for zebrafish [31], for mice [47]). Based on the literature, at least two alternative explanations for the altered (horizontal and vertical) spatial distribution patterns have to be considered: (1) spatial disorientation as might be indicated by poor spatial memory performance in zebrafish treated with MK-801 [30] and (2) increased occurrence of restrictive repetitive behaviors (which is one of the diagnostic criteria for ASD [48]) as is indicated by increased circling behavior in both mice [23] and zebrafish [32] and increased erratic movements in zebrafish [31] treated with the drug. Stereotypic behaviors have not been extensively explored in zebrafish and at this point it is difficult to predict under what conditions they would result in spatial redistribution. A promising approach is to analyze the spatial and kinematic patterns in more detail. For instance, as one of the reviewers suggested, it might be interesting to look at homebase formation [49], [50]. In this study, we did not systematically investigate this aspect of the MK-801 zebrafish, since homebase behavior is not easy to assess in shoals of fish and because it is not directly related to the new paradigm presented here, which focuses on social cohesion. However, preliminarily we looked at spatial allocation preferences (based on a 1000-cell partitioning of the observation container) in representative zebrafish shoals of experiments 1 and 2 (Fig. S1 and S2). It should be pointed out that spatial allocation is not identical with homebase behavior, since the latter also takes into account traveled distance in and number of visits to the spatial zones (or cells) under consideration [49], [50]. The examples (Fig. S1) suggest that for the homogeneous groups (in experiment 1), the spatial allocation preference, which is usually very pronounced in control zebrafish, disappears for 2 µM and a vertical allocation preference appears for 5 µM as attraction to the water surface increases, however, without emergence of a more limited region that could function as homebase. One problem is that the 1000-cell allocation of a shoal represents the averaged data for all its four members. Since social cohesion was very weak in zebrafish treated with higher doses of MK-801, the prediction would be that homebase behavior, if indeed preserved, would result in separate locations for the four zebrafish (unless there is a common environmental attractor). Since the software does not consistently identify the individual zebrafish (i.e. tag swapping occurs), such an analysis is difficult to perform. Also note that spatial allocation could not only be affected by the presence of homebases but also by specific locations that are characterized by increased path tortuosity (or so-called ‘knots’, as described in mice [51]). For the heterogeneous groups (Fig. S2), the interpretation problems are even more complex since we would have to discern the drug-treated zebrafish from the control zebrafish. Although these topics are important for the interpretation of the behavioral effects of MK-801 and deserve much more research, in the present study they are not of major interest because the focus is on the effect of the drug on social engagement as means to evaluate the introduced alternative behavioral paradigm.

Testing MK-801 in the new heterogeneous-shoaling paradigm resulted in two interesting findings. First, treating one member of the quadruplets with 5 µM MK-801 was sufficient to obtain a strong decrease of social cohesion for the entire shoal. This was probably due to the fact that the three untreated zebrafish changed their behaviors when faced with the altered behavior of the drug-treated zebrafish. This interpretation was supported by the increase of all four NND-components and further confirmed by visual observations. However, significantly more research is needed to elucidate the changes in social dynamics of the heterogeneous shoals. Second, non-social behaviors such as distance from bottom, travel distance and distribution over the four quadrants seem to be less affected than social cohesion by the presence of one zebrafish treated with MK-801. This dissociation between social and non-social behaviors by the new paradigm might be of value when the objective is to isolate social effects of a drug or mutation.

## Supporting Information

Figure S1 **Spatial allocation in the 1000-cell system for homogeneous shoals in experiment 1.** For every dose one representative example of the 1000-cell spatial allocation is presented. The observation container was virtually divided into 10×10×10 cells, each 25×25×13.5 mm (l×w×h). Each cell is labeled by the time the fish spent in that cell expressed as percentage of the total observation time and averaged over the four fish of the quadruplet. The diagrams (top, side and front view) present the orthogonal projections such that the ten cells in the not-presented dimensions are summated. Thus, per projection 100 summary cells (or columns) are presented. Assuming homogenous spatial distribution, each column would have 1% occupancy. Note the shift of spatial allocation with increasing dose.
(TIF)

Figure S2 **Spatial allocation in the 1000-cell system for heterogeneous shoals in experiment 2.** For both groups two representative examples are shown. Coding is similar as in Fig. S1. For the experimental group, a clear-cut pattern could not be established.
(TIF)
