# Supplementary figures and images for: Assessing Social Engagement in Heterogeneous Groups of Zebrafish: A New Paradigm for Autism-Like Behavioral Responses
Source: PLoS One. 2013 Oct 8;8(10):e75955. doi: 10.1371/journal.pone.0075955 (PMC3792997; doi:10.1371/journal.pone.0075955)

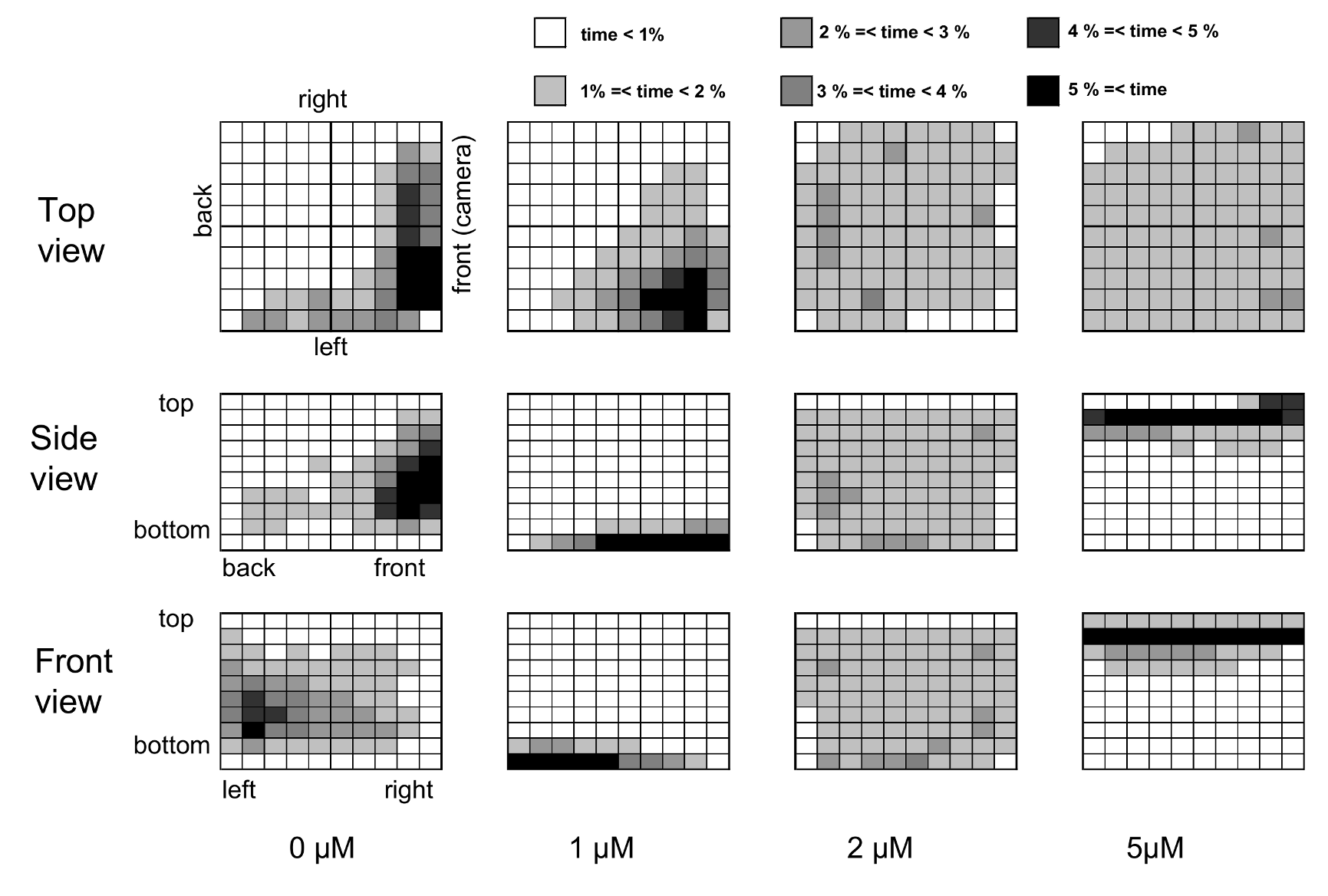

Supplement: Figure S1 — Spatial allocation in the 1000-cell system for homogeneous shoals in experiment 1. For every dose one representative example of the 1000-cell spatial allocation is presented. The observation container was virtually divided into 10×10×10 cells, each 25×25×13.5 mm (l×w×h). Each cell is labeled by the time the fish spent in that cell expressed as percentage of the total observation time and averaged over the four fish of the quadruplet. The diagrams (top, side and front view) present the orthogonal projections such that the ten cells in the not-presented dimensions are summated. Thus, per projection 100 summary cells (or columns) are presented. Assuming homogenous spatial distribution, each column would have 1% occupancy. Note the shift of spatial allocation with increasing dose. (TIF) [file pone.0075955.s001.tif]

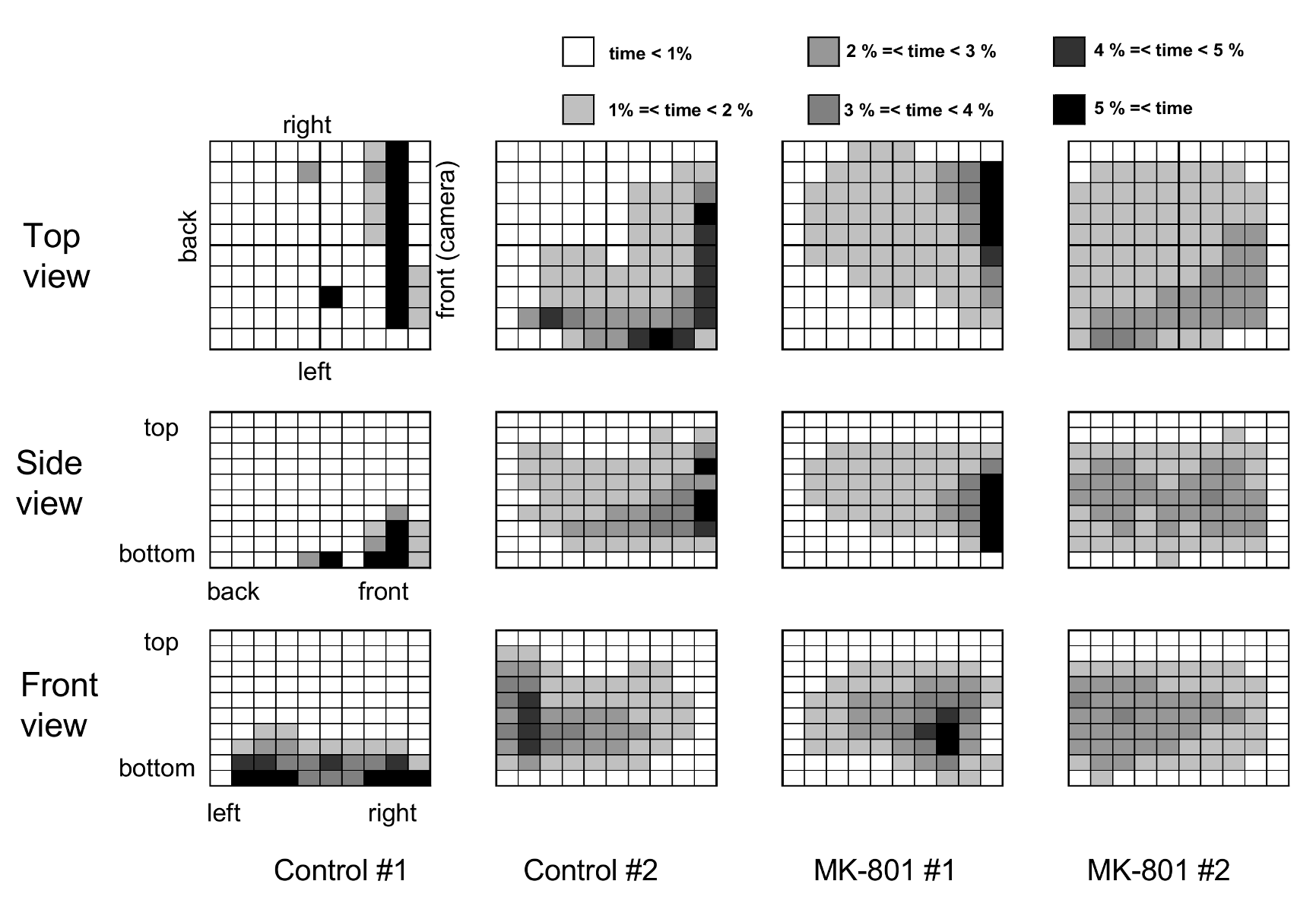

Supplement: Figure S2 — Spatial allocation in the 1000-cell system for heterogeneous shoals in experiment 2. For both groups two representative examples are shown. Coding is similar as in Fig. S1. For the experimental group, a clear-cut pattern could not be established. (TIF) [file pone.0075955.s002.tif]
